# Ultrafast temporal evolution of interatomic Coulombic decay in NeKr dimers†

**DOI:** 10.1039/d1sc04630f

**Published:** 2021-12-29

**Authors:** F. Trinter, T. Miteva, M. Weller, A. Hartung, M. Richter, J. B. Williams, A. Gatton, B. Gaire, J. Sartor, A. L. Landers, B. Berry, I. Ben-Itzhak, N. Sisourat, V. Stumpf, K. Gokhberg, R. Dörner, T. Jahnke, T. Weber

**Affiliations:** Institut für Kernphysik, Goethe-Universität 60438 Frankfurt am Main Germany trinter@atom.uni-frankfurt.de; Molecular Physics, Fritz-Haber-Institut der Max-Planck-Gesellschaft 14195 Berlin Germany; Laboratoire de Chimie Physique Matière et Rayonnement, UMR 7614, Sorbonne Université, CNRS 75005 Paris France; Lawrence Berkeley National Laboratory, Chemical Sciences Division Berkeley California 94720 USA tweber@lbl.gov; Department of Physics, University of Nevada Reno Nevada 89557 USA; Department of Physics, Auburn University Auburn Alabama 36849 USA; J. R. Macdonald Laboratory, Department of Physics, Kansas State University Manhattan Kansas 66506 USA; Theoretische Chemie, Physikalisch-Chemisches Institut, Universität Heidelberg 69120 Heidelberg Germany; European XFEL GmbH 22869 Schenefeld Germany till.jahnke@xfel.eu

## Abstract

We investigate interatomic Coulombic decay in NeKr dimers after neon inner-valence photoionization [Ne^+^(2s^−1^)] using a synchrotron light source. We measure with high energy resolution the two singly charged ions of the Coulomb-exploding dimer dication and the photoelectron in coincidence. By carefully tracing the post-collision interaction between the photoelectron and the emitted ICD electron we are able to probe the temporal evolution of the state as it decays. Although the ionizing light pulses are 80 picoseconds long, we determine the lifetime of the intermediate dimer cation state and visualize the contraction of the nuclear structure on the femtosecond time scale.

## Abstract 2

We investigate the time-resolved dynamics of interatomic Coulombic decay in NeKr dimers after neon inner-valence photoionization using a synchrotron light source.

## Graphical abstract

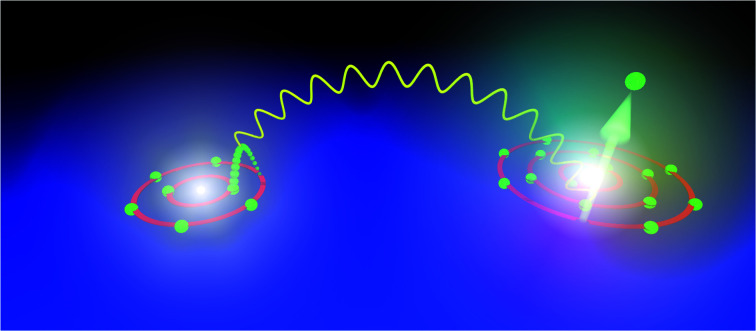

## Introduction

In the last two decades, interatomic (or intermolecular) Coulombic decay (ICD) has been identified as a very common relaxation pathway in nature that occurs after electronically exciting a weakly bound system (*e.g.*, van der Waals or hydrogen bonded compounds) by ion, electron, or photon impact, respectively^1–20^ (see ref. 21–23 for reviews). ICD occurs when an excited system, which is embedded in a chemical environment of loosely bound neighboring atoms or molecules, relaxes by transferring the excess excitation energy to a neighboring atom or molecule. This energy release typically happens on a femtosecond (fs) time scale and leads to the ejection of a low-energy secondary (ICD) electron from a neighboring site. The ICD electron energy is typically on the order of a few electron volts. If the initial excitation consists of an ionization of the cluster compound, the cation formation, occurring as a result of the decay, initiates a Coulomb explosion of the system and results in the fission of the weak bond. Beside breaking the bond, the low-energy electrons ejected by the ICD process are prone to induce biological damage such as DNA-strand breaks in secondary reactions, and the cationic fragments can react with surrounding biomolecules, causing further damage to the biological system.^24,25^ On the positive side, ICD is expected to play an important role as a repair mechanism for DNA enzymes.^26^ ICD processes, hence, represent a fundamentally important class of ionization mechanisms in photochemistry and are of multidisciplinary relevance, for example, in weakly bound matter of biological systems.

In this article, we examine the temporal evolution of an IC-decaying system. The ICD lifetime of a dimer is rooted in the nature of the transient electronically excited dimer states, *i.e.*, the charge state, the internuclear distance, as well as the steepness and variety of the accessible potential energy curves (PECs). Typical time scales of ICD are on the order of several tens to a few hundreds of femtoseconds. Neon dimers, for example, decay within (150 ± 50) fs after inner-valence ionization as demonstrated in a pump-probe experiment by Schnorr *et al.*^27^ In exceptional cases such as HeHe dimers, ICD takes place over a rather long time period, which is greater than 2000 fs measured with an internal clock^28^ (see also ref. 29 for a review). In the present work, we employ the same experimental approach as in the aforementioned HeHe case^28^ to NeKr dimers. NeKr dimers represent an excellent target to study ICD dynamics on very short time scales, as they exhibit a much faster decay of the transient cationic state than HeHe dimers. This is mainly because of the more than 10 times smaller bond length of the initial neutral state and the steeper gradient of the interatomic potential of the decaying cationic state. As a consequence, the ICD rates in the Franck–Condon region are larger, and it takes the dimer less time to contract to shorter internuclear distances such that ICD rates increase even further.^30^ The NeKr dimer also has a more complex vibrational structure in the decaying state than HeHe, where several densely spaced vibrationally excited states of the transient Ne^+^(2s^−1^)Kr cation are populated so that a vibrational wave packet of overlapping resonance states is formed.

Instead of applying a pump-probe scheme, we retrieve the timing of the ICD event indirectly using an internal clocking procedure based on post-collision interaction (PCI)^31–35^ between the emitted photoelectron and ICD electron. This approach has been introduced in ref. 28 and is termed PCI-streaking. In our experiment, the NeKr dimer absorbs a single photon expelling a very slow photoelectron that starts to leave the singly ionized dimer. As the ICD happens, the photoelectron is “overtaken” by the faster ICD electron and pulled back by the Coulomb force of the newly created doubly charged dimer ion. This results in a decrease of the kinetic energy of the emerging photoelectron, whereas the kinetic energy of the ICD electron increases. As demonstrated in ref. 28, the amount of deceleration of the photoelectron depends on the emission time of the ICD electron, which emerges from the ion and “overtakes” the slower photoelectron, changing the attractive cation potential to a dication one. Thus, by measuring the energy shift of the photoelectron, information on the time delay between the excitation and decay can be retrieved. For very short decay times and for photoelectron energies very close to the ionization threshold, the energy loss can be so large that the electron is recaptured to a Rydberg state.^36^

In the following, we report on a detailed experimental investigation of ICD in NeKr dimers after Ne^+^(2s^−1^) photoionization, where we apply the method of PCI-streaking and measure the energy shift of the slow photoelectron in order to infer the time of the decay. The reaction proceeds as follows:

1 *γ*(48.60 eV/48.68 eV) + NeKr → Ne^+^(2s^−1^)Kr + e_photo_ → Ne^+^ + Kr^+^ + e_photo_ + e_ICD_.

As in our investigation on HeHe dimers,^28^ we recorded two datasets, employing different photon energies for the excitation of the IC-decaying state, namely 48.60 eV and 48.68 eV. In the absence of PCI, these energies would correspond to emitting Ne(2s) photoelectrons with 120 and 200 meV kinetic energy, respectively, since the Ne(2s^−1^) ionization threshold lies at 48.48 eV. With the two different photoelectron energies, we obtain one dataset with high time resolution (low photoelectron energy of 120 meV) but truncated access to short time scales due to the relatively high loss to electron recapture, and another dataset with moderate time resolution (high photoelectron energy of 200 meV) but rather little loss to electron recapture and, hence, access to shorter time scales.

## Theory

Experimental observables such as the squared norm of the vibrational wave packet and the mean internuclear distance of the decaying electronic state as a function of time can be obtained theoretically. To this end, we apply the Born–Oppenheimer approximation to separate the electronic and nuclear degrees of freedom and first obtain the electronic properties of the dimer, *i.e.*, the adiabatic PECs and the ICD width. These properties are used in turn to solve the time-dependent Schrödinger equation for the nuclear motion, as we explain in more detail below. The observables are then extracted from the resulting nuclear wavefunction and compared with the results of the experiment.

**Fig. 1:**
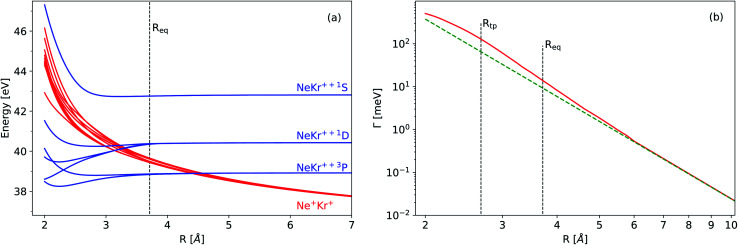
(a) Potential energy curves of the final states of the decay of Ne^+^(2s^−1^)Kr (see text for the computational details). The blue and red diabatic curves correspond to the doubly charged one-site Ne–Kr^++^ states and to the doubly charged two-site repulsive Ne^+^–Kr^+^ states, respectively. (b) Total electronic decay width for Ne^+^(2s^−1^)Kr ^2^S (red curve). The vertical lines correspond to the classical turning point (*R*_tp_) and to the equilibrium distance of the ground vibrational state (*R*_eq_) of Ne^+^(2s^−1^)Kr ^2^S. The dashed green line shows a 1/*R*^6^ dependence to guide the eye.

**Fig. 2:**
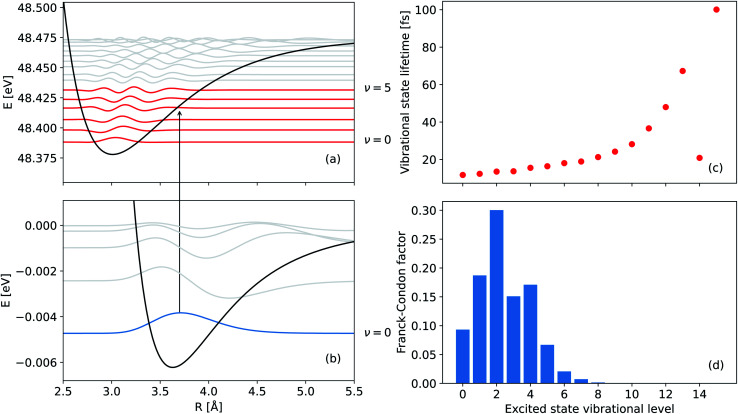
PECs of (a) the singly ionized Ne^+^(2s^−1^)Kr state and (b) the ground electronic state of NeKr. The vibrational levels supported by these states are also shown. The zeroth vibrational state of the ground state as well as the real parts of the vibrational wave functions of the singly ionized state, which have the highest Franck–Condon factors are depicted in blue and red, respectively. (c) Vibrationally resolved lifetimes (fs) of the 16 vibrational states, supported by the Ne^+^(2s^−1^)Kr PEC, and (d) their respective Franck–Condon factors for the transition from the vibrational ground state of NeKr to the singly ionized state of Ne^+^(2s^−1^)Kr. The lifetimes and the Franck–Condon factors were computed by diagonalizing the complex Hamiltonian, including the lifetime broadening of the decaying state (see text for details).

The neutral NeKr dimer in its electronic ground state is weakly bound by van der Waals forces. The binding energy and equilibrium distance, obtained in highly accurate coupled-cluster calculations,^37^ are 5.9 meV and 3.67 Å, respectively. The inner-valence ionization of Ne and the following interatomic decay populate the Ne^+^(2s^−1^)Kr decaying state and the Ne^+^Kr^+^ final states. We computed the respective PECs of NeKr [see Fig. 1(a) and 2(a)].

The initial state (ground state) PEC was taken from ref. 38. The mean internuclear distance and the binding energy of this potential are 3.62 Å and 6.2 meV, respectively. These values are in good agreement with the value of the mean internuclear distance between the atoms of the NeKr dimer, which were predicted to be (3.58 ± 0.11) Å according to ref. 39 (calculated in 1977) and 3.67 Å according to ref. 40 (calculated in 2020). The binding energy of NeKr dimers also agrees well with the value of 6.1 meV reported in ref. 41 and 42, while homonuclear KrKr dimers are bound by 17.3 meV according to ref. 42 and 43, and NeNe dimers are bound by 3 meV according to ref. 44 and 45.

The PEC of the decaying state was computed using the Green's function Algebraic Diagrammatic Construction [ADC(3)] method,^46,47^ employing the aug-cc-pCVQZ basis set on Ne and the aug-cc-pVQZ basis set on Kr.^48^ The depth of the potential minimum was further adjusted in order to correctly reproduce the experimental photoelectron spectrum (see below). The adiabatic PECs of the final states were obtained by the two-hole propagator ADC(2) method,^49^ using the aug-cc-pVQZ basis set on both atoms. In this calculation, the Ne 1s and Kr 1s, 2s, 2p, 3s, 3p, and 3d atomic orbitals were kept frozen. In the region from 3.5 Å to 2.9 Å, there are several avoided crossings between the adiabatic states, which correspond to the Ne^+^–Kr^+^ and Ne–Kr^++^ channels [note that the diabatic states are shown in Fig. 1(a)]. Decay to Ne–Kr^++^ states is often called the electron transfer mediated decay (ETMD) channel (see, *e.g.*, ref. 50 and 51). In NeKr dimers, the direct population of these states *via* the ETMD process is suppressed by concurrent ICD. However, these states can be populated due to their non-adiabatic coupling with the ICD channels, if the decay events take place at *R* ≲ 3.5 Å. Nevertheless, due to the very short ICD lifetime, the decay occurs mostly around the equilibrium internuclear distance *R*_eq_. We therefore neglect the population and nuclear dynamics of the ETMD channel in our calculations. Furthermore, the coupling between ETMD and ICD channels due to non-adiabatic effects is negligible. We therefore employ the diabatic PECs, shown in Fig. 1(a), corresponding to ICD channels (red lines) in the nuclear dynamics simulations. These diabatic PECs are purely Coulombic at large interatomic distances, as expected for the strong repulsion between the two final ions.

The electronic decay width of the metastable Ne^+^(2s^−1^)Kr state was computed by the Fano-ADC-Stieltjes approach,^52,53^ where the transient and final states were described at the ADC(2) extended level. The cc-pVQZ basis set was used on both atoms. The basis set was augmented by four s-, four p-, four d-, and one f-Gaussian-type functions with continuum-like Kaufmann exponents^54^ in the case of Ne, and six s-, six p-, six d-, and four f-functions in the case of Kr. Additional five s-, five p-, and five d-functions were added at the mid-bond location. The diffuse basis functions were used to improve the description of the discretized electronic continuum states. Fig. 1(b) shows the calculated total electronic decay width for the Ne^+^(2s^−1^)Kr photoionization as a function of the internuclear distance. Note that at large interatomic distances the ICD width scales as 1/*R*^6^, as expected by the virtual-photon approximation.^30,55^

The nuclear dynamics of the ICD process can be described by propagating wave packets on the PECs of the initial and final ICD states. In our calculations, we assume that the ionization step is instantaneous (sudden approximation), and we neglect the polarization caused by the slow photoelectron^56,57^ and the interactions between the initial and final ICD states similar to previous calculations of ICD dynamics.^58,59^ Following these assumptions, the time evolution of the wave packets of the decaying and final states can be described by the following set of time-dependent Schrödinger equations:

2 i|*Ψ̇*_d_(*t*)〉 = (*T̂*_N_ + *V̂*_d_ − i*Γ̂*_d_(*R*)/2)|*Ψ*_d_(*t*)〉

3
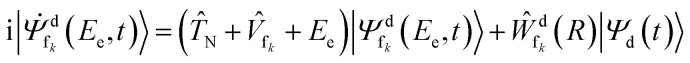
Here *T̂*_N_ is the kinetic energy of the nuclei, where we assume that an angular momentum of *J* = 0 is an adequate choice for the NeKr dimer, since the experimental target is in its rovibrational ground state (see below). *V̂*_d_ is represented by the potential energy curve of the decaying state, and *V̂*_f_*k*__ are represented by the diabatic repulsive PECs of the final states, respectively. *Ψ*_d_(*t*) and 
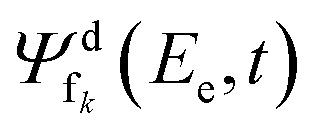
 are the wave packets of the decaying and final states. The final-state nuclear wave packet depends on the kinetic energy of the ICD electron *E*_e_. The transition amplitude 
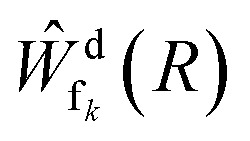
 is related to the partial decay width of state d to state f_*k*_ as 
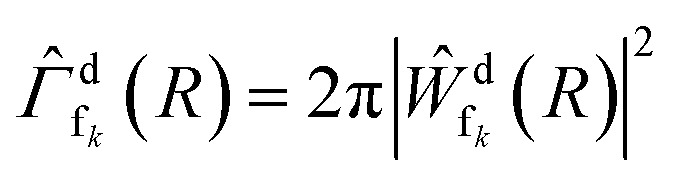
. Assuming an instantaneous ionization, the initial condition for eqn (2) was taken to be the eigenfunction of the zeroth vibrational level of the ground electronic state, whereas at time *t* = 0, the initial conditions for the final state in eqn (3) were set to 0. The set of equations was solved separately for each electronic final state ^1,3^Σ^+^ (*x*2), ^1,3^Σ^−^, ^1,3^Π (*x*2), and ^1,3^Δ.

The kinetic energy release (KER) spectrum was computed as described in ref. 61, such that the partial spectrum, which arises due to the decay into channel f_*k*_, is given by4
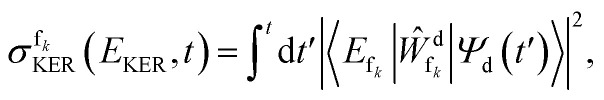
where |*E*_f_k__〉 is an eigenfunction of the Hamiltonian *Ĥ*_f_k__ = *T̂*_*N*_ + *V̂*_f_k__ with energy *E*_f_k__ = *E*_KER_ + *V*_f_k__(∞). The total KER spectrum was evaluated as a weighted sum of the partial spectra, corresponding to the final electronic states, *i.e.*, as5
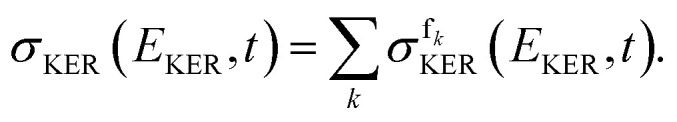
The above equation describes the cumulative KER spectrum up to a specific point in time. In order to compute the KER spectrum for a specific time interval, the difference between two consecutive time steps has to be considered.

The vibrational states supported by the bound electronic PECs of the ground and singly ionized states (see Fig. 2) were computed by diagonalizing the respective nuclear Hamiltonian matrices (*T̂*_N_ + *V̂*_i_) and (*T̂*_N_ + *V̂*_d_ − *Γ̂*_d_(*R*)/2). The latter were represented on a uniformly spaced grid of 1024 points for distances spanning the range from 2.0 Å to 12.23 Å, using a discrete variable representation (sine DVR) basis set.^62^ In the case of the decaying state with a decay width *Γ̂*_d_, a complex eigenvalue problem, accounting for the lifetime broadening, is considered

6 (*T̂*_N_ + *V̂*_d_ − i*Γ̂*_d_(*R*)/2)|*χ*^d^_*ν*_〉 = *ε*^d^_*ν*_|*χ*^d^_*ν*_〉

where *ε*^d^_*ν*_ and *χ*^d^_*ν*_ are the complex vibrational eigenvalues and eigenfunctions of the decaying state, respectively. The vibrational lifetimes shown in Fig. 2 are evaluated as the inverse of the imaginary part of the vibrational eigenvalues 2Im(*ε*^d^_*ν*_). We found that the ground state PEC supports five vibrational states, while 16 vibrational levels can be accommodated by the PEC of the decaying state [see Fig. 2(a)]. The respective vibrational wavefunctions were used to compute the Franck–Condon factors for the transitions between the vibrational ground state in the initial electronic state of NeKr and the vibrational states in the decaying state [see Fig. 2(b)].

The theoretical approach employed in this work accounts only for the ICD process, thus neglecting the PCI effect. While it was shown that the correlation between the photoelectron and the Auger electron may affect the spectra in the Auger decay of atoms,^60^ it should be noted that the two-step approach used in this work has been successfully applied to describe the experimental observations in a related experiment on He dimers.^36^

## Experiment

The measurements have been performed at the undulator beamline 10.0.1.3 at the synchrotron-radiation facility Advanced Light Source (Berkeley, CA) during two-bunch mode operation using reaction microscopy, also known as COLd Target Recoil Ion Momentum Spectroscopy (COLTRIMS).^63–65^ The NeKr dimers were produced by expanding a 97% and 3% mixture of Ne and Kr, respectively, through a cooled nozzle (30 μm diameter) at a temperature of 140 K and a driving pressure of 5.5 bar. This resulted in a fraction of <1% NeKr dimers in the gas jet. Given the internal jet temperature of <10 K, corresponding to an energy of <1 meV (taking into account the momentum uncertainty) and the energy spacing between the *ν* = 0 and *ν* = 1 vibrational states [see Fig. 2(b)], we can conclude that the NeKr dimers in the target gas jet are in the vibrational ground state. The supersonic gas jet passed through two skimmers (0.3 mm and 0.5 mm diameter) and was crossed with the linearly polarized photon beam at right angle inside the 3D momentum imaging spectrometer, which was equipped with position- and time-sensitive multi-hit capable detectors for registering electrons and ions in coincidence.

In this experiment, the Ne^+^ and Kr^+^ ions and the very low-energy photoelectron occurring in the final state were measured in coincidence (the ICD electron was not directly detected). From the measured photoelectron energy, the decay time was inferred by making use of the PCI between photoelectrons, ICD electrons, and dimer dications, as explained in the introduction. This measurement of the temporal evolution of the state *via* the internal PCI clock method requires a narrow photon bandwidth and a high energy resolution measurement of the low-energy photoelectron. The latter was achieved using a very weak acceleration electric field of only 1.2 V cm^−1^ in the spectrometer. No parallel magnetic field, which is often crucial to ensure full solid detection angle for high-energy electrons in COLTRIMS measurements, was required due to the low energy of the photoelectrons. Ions and electrons created in the interaction region were guided onto two 120 and 80 mm micro-channel-plate detectors with delay-line position readout,^66^ respectively. The spectrometer consisted of an electron arm with a 70 mm acceleration section followed by a 140 mm drift region for time-of-flight focusing in a Wiley–McLaren geometry. The ion detection arm consisted of a 36 mm acceleration region. The times-of-flight and positions of impact of the ions and photoelectrons were measured in coincidence on a shot-by-shot basis.

A simple simulation of the particle trajectories in the spectrometer yields the following results in terms of momentum resolution for the charged particles, which were imaged by the electric field, as shown in Table 1. According to these numbers and the measured KER of Ne dimers (that are generated in our gas jet as well), which we can compare to ref. 4, we conclude that our energy resolution is better than ΔKER ≈ ±160 meV and corresponds to a resolving power of KER/(ΔKER) ≈ 24. This KER resolution translates to an error in the extracted interatomic distance *R* of Δ*R* ≈ ±0.15 Å. From several photo-effect calibration measurements of helium (10, 30, 60, 120, 150, 200, and 250 meV above the ionization threshold), we extracted the photoelectron energy resolving power to be *E*/Δ*E* ≈ 10.

**Table 1:** Mean momentum resolution (FWHM) in the *x*-, *y*-, and *z*-directions for electrons, Ne^+^ ions, and Kr^+^ ions

	Electrons	Ne^+^ ions	Kr^+^ ions
*x*-direction (photon beam)	0.001 a.u.	0.318 a.u.	0.652 a.u.
*y*-direction (gas jet)	0.001 a.u.	0.318 a.u.	0.652 a.u.
*z*-direction (time-of-flight)	0.006 a.u.	0.006 a.u.	0.006 a.u.

**Fig. 3:**
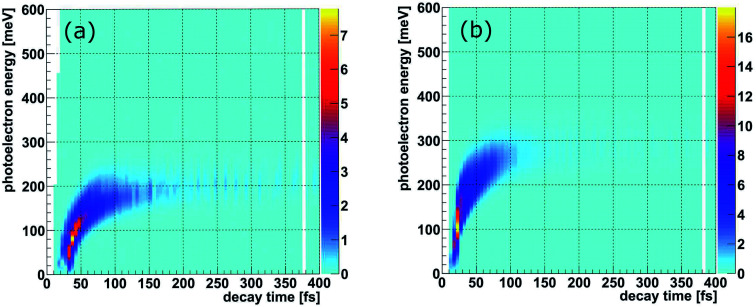
Simulated kinetic energy (meV) of the photoelectron resulting from the ICD process of NeKr dimers as a function of the decay time (fs) for (a) 48.60 eV and for (b) 48.68 eV photon energy. Here, all vibrational states, including their Franck–Condon factors, were used. Additionally, an approximated experimental photoelectron energy resolution of Δ*E*/*E* ≈ 10% (see text) was convoluted.

The PCI clock method described in the introduction and in ref. 28 yields a one-to-one mapping of the decay time to the photoelectron energy loss *E*(*t*) (see Fig. 3(b) of ref. 28). To apply this scheme to the present experiment, two additional effects have to be included: firstly, there are 16 vibrational states of the Ne^+^(2s^−1^)Kr cation yielding 16 functions *E*_*ν*_(*t*), one for each vibrational state *ν*. The relative weight of each of these contributions to the final energy distribution is represented by the Franck–Condon factors between the NeKr ground state [with *J* = 0, see Fig. 2(d)] and the respective ionic Ne^+^(2s^−1^)Kr vibrational state [Fig. 2(a) and (b)]. Secondly, each of the functions *E*_*ν*_(*t*) has to be convoluted with a Gaussian energy distribution, mimicking the finite photoelectron energy resolution of our experiment, which can be approximated being constant for the present very low photoelectron energies. This yields a mapping of each measured photoelectron energy to a distribution of decay times, which is shown in Fig. 3. It is important to stress that this map is different from an unequivocal correlation function between decay time and photoelectron energy: in this map, every decay time depends on the whole range of measured photoelectron energies (and *vice versa*), which is extracted by projections on the respective axis for selected small slices (*e.g.*, ±10 fs) of the other axis. Using these maps, we can relate the measured KER (which is connected to the internuclear distance at the instant of the decay, see below) to the decay time. This is possible because we measure the fragment ions in coincidence with the photoelectrons on a shot-by-shot basis. Hence, by correlating the ion energies and the KER, respectively, with the photoelectron energy, the nuclear motion can be coupled to the inferred decay times from the PCI-streaking scheme. The details are given in the ESI.†

## Results

**Fig. 4:**
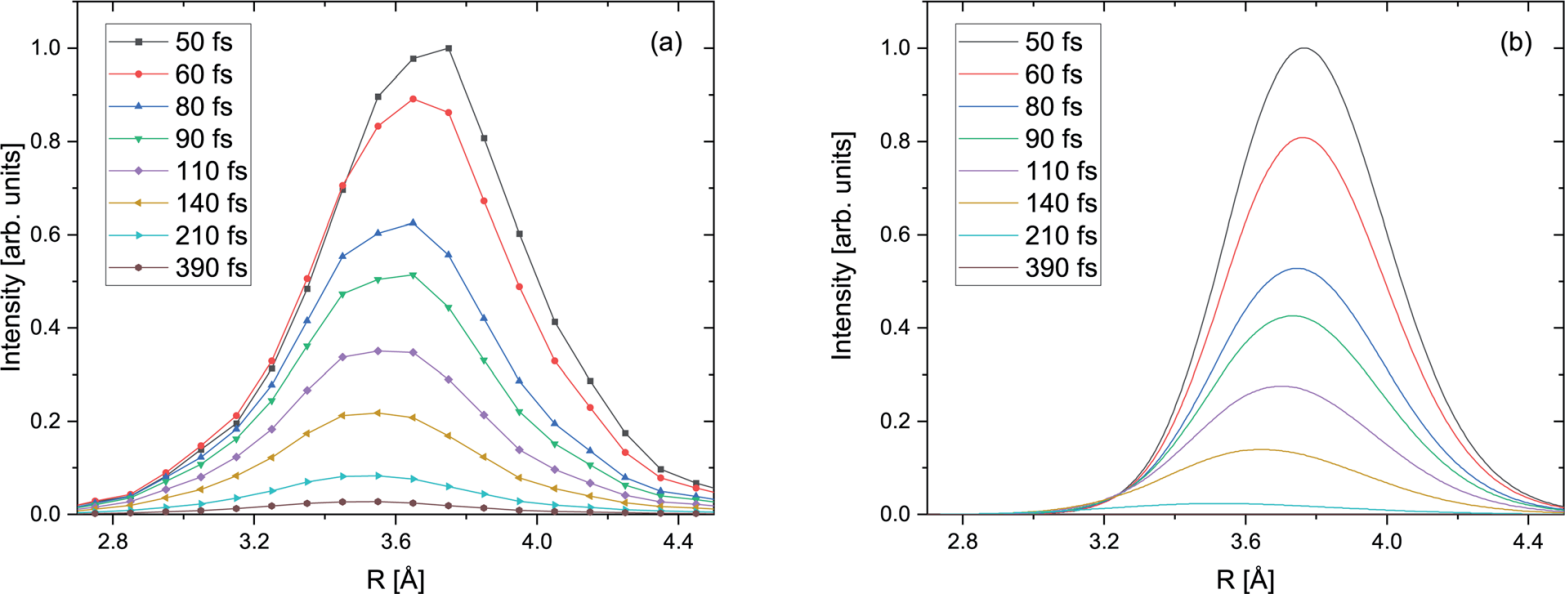
(a) Measured yield of the ICD process as a function of the internuclear distance *R* of the NeKr dimer for 48.60 eV photon energy for select inferred decay times (±10 fs), as indicated in the legend *via* the PCI-streaking scheme (see text). For the complex conversion from the measured photoelectron energy to the decay time we made use of Fig. 3. As the fragment ions are measured in coincidence with the photoelectrons, as shown in Fig. SI1 in the ESI,† the inferred decay times are correlated to the *R* distributions *via* projections of Fig. SI2† onto the KER ∼ 1/*R* axis. A trend towards smaller internuclear distances *R* at longer decay times is clearly visible. The error in the retrieved *R* is estimated to be ±0.15 Å. (b) Calculated yield of the ICD process as a function of the internuclear distance *R* of the NeKr dimer for select decay times (see legend).

Within the reflection approximation,^67^ the measured KER of the ions is related to the internuclear distance *R* of the atoms in the dimer at the instant of the Coulomb explosion (in atomic units) *via* KER ∼ 1/*R*. This is justified as the diabatic PECs are purely Coulombic in the relevant region of large internuclear distances, as described in the theory section. Employing our PCI-streaking clocking method, this allows us to extract the *R* distribution from the ion coincidence data as a function of real-time. The corresponding results are shown in Fig. 4(a) for the photon energy of 48.60 eV. Note that the internuclear distance spectra for single time steps were not normalized to their integral nor to their maximum. They were staggered according to their increasing time steps from top to bottom, *i.e.*, they depict the decays for small time intervals (±10 fs) as a function of the internuclear distance. The time steps were chosen to increase with longer decay times in order to compensate for the fact that most of the nuclear dynamics takes place at shorter decay times and relatively few decays occur at longer times. The peak values of the internuclear-distance distributions decrease step-wise from 3.8 Å to 3.5 Å with time. Evidently, we resolve the contraction of the Ne^+^(2s^−1^)Kr dimer cation in increments of 0.3 Å. We also see that the excited dimer decays completely within the first 250 fs.

The distributions presented in Fig. 4(a) were generated by integrating the KER spectra, which were used to convert the energy to interatomic distance, over time intervals of ±10 fs for each individual time step. They are in good agreement with our calculations, shown in Fig. 4(b). We note again that the nuclear dynamics calculations exclusively account for ICD, *i.e.*, that the electron transfer mediated decay (ETMD) channel is assumed to be closed.

**Fig. 5:**
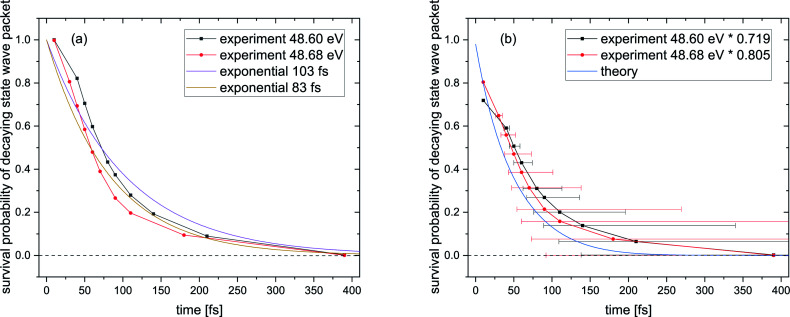
(a) Survival probability of the decaying state wave packet of the intermediate Ne^+^(2s^−1^)Kr cation state as a function of the inferred decay time from the PCI-streaking scheme (see text). The black and red data points depict the experimental results for a photon energy of 48.60 eV (black) and a photon energy of 48.68 eV (red). Here, the loss due to the photoelectron recapture is not yet corrected for. (b) The same data after correcting for the photoelectron recapture process, by using the approximation of an exponential decay with lifetimes of 103 fs for the 48.60 eV and 83 fs for the 48.68 eV experiment. This approach is represented by the purple and brown curves in panel (a). The asymmetric error bars in panel (b) stem from the non-linear conversion of the measured photoelectron energy to the decay time. The blue curve in panel (b) represents the theoretical results, see text for details.

Fig. 5(a) shows the survival probability, *i.e.*, the squared norm of the decaying state wave packet of the excited Ne^+^(2s^−1^)Kr cation state as a function of the decay time (see also ref. 28). For certain times *t*, this squared norm of the decaying state wave packet as a function of time is computed as 1 − *A*(*t*)/*A*_total_, where *A*(*t*) is the area below the KER spectrum at time *t*, and *A*_total_ is the area below the KER spectrum at *t* → ∞, taken from the KER *vs.* time maps in Fig. SI2 in the ESI.† The blue curve in Fig. 5(a) represents the *ab initio* calculation of the squared norm of the wave packet of the decaying state as a function of time: 〈*Ψ*_d_(*t*)|*Ψ*_d_(*t*)〉. The black and red curves represent the experimental results for photon energies of 48.60 eV and 48.68 eV, respectively. The experimental and theoretical values show only moderate agreement. Furthermore, even the two measured curves exhibit some disagreement, whereas they were expected to follow the same ICD dynamics. The reason for this difference is the fact that the probability for recapture of the photoelectron^36^ strongly depends on the photoelectron energy: the lower the photoelectron energy, the more likely a recapture can occur, which consequently truncates the decay-time measurement at a certain minimal time value.

It is hence crucial to know the fraction of photoelectrons that were recaptured for each photon energy at each kinetic energy of the post-interacting particles. We obtain this fraction by fitting the data in Fig. 5(a), assuming an exponential decay law. In general, ICD does not follow an exponential decay law [exp(−*t*/*τ*)], because it is driven by the interplay of electron transitions and nuclear motion, and, as shown in Fig. 1(b), the decay rates dramatically depend on the internuclear distance. Nevertheless, for NeKr an exponential decay fits our experimental data very well. To this end, an exponential function exp(−*t*/(103 fs)) (*R*^2^ = 0.946) for the 48.60 eV photon energy measurement, and exp(−*t*/(83 fs)) (*R*^2^ = 0.953) for the 48.68 eV measurement, fit the data, as represented by the purple and brown curves in Fig. 5(a). The fit function was constrained to start at a squared norm of the decaying state wave packet of 1 at time zero and only included the lifetime *τ* as a fit parameter. This yielded reasonable *R*^2^ values and returned correction factors based on the lifetimes *τ* for both measurements, which take into account the amount of recapture in the following way. From Fig. 3(a) and (b) we can extract the shortest decay times accessible to our PCI measurement scheme as the cut-off decay time, *i.e.*, the minimum value of the abscissa of these 2D spectra. For a photon energy of 48.68 eV, this lower time limit is 18 fs (using the mean value of the Franck–Condon factors of all vibrational states). For shorter times than this cut-off value no photoelectron and corresponding ion could be observed in coincidence in the measurements due to the recapture process losses. According to this minimum value, *t*_min_, the exponential decay function exp(−*t*_min_/(83 fs)) yields a correction factor of exp(−18/83) = 0.805. This number means that for a 48.68 eV photon energy only 80.5% of all possible photoelectron-ion events could be measured in coincidence. For a photon energy of 48.60 eV, the lower time limit is 34 fs. In this case a correction factor of exp(−*t*_min_/(103 fs)) = exp(−34/103) = 0.719 is determined. *Vice versa* it is possible to deduce the percentage of recapture processes and correct the time-resolved decay curves shown in Fig. 5(a) by normalizing the decay-curve data points with the determined correction factors. The result of this correction is shown in Fig. 5(b).

The survival probability was computed as the squared norm of the decaying state wave packet whose time dependence was obtained by solving eqn (2), *i.e.*, as 〈*Ψ*_d_(*t*)|*Ψ*_d_(*t*)〉. After implementing the recapture corrections in the analysis of the experimental data, the agreement between the two measurements and theory is satisfying. The employed PCI-streaking method and therefore the non-linear conversion from the measured photoelectron energy to the decay time leads to relatively large experimental error bars. Within these error bars, the results for the exponential fits to the experimental data are given above, while an exponential fit to the theoretical norm of the decaying state wave packet yields a lifetime of 48 fs. This theoretical number fits the measured lifetimes satisfyingly well in Fig. 5(b) (a zoomed-in version is presented in Fig. SI5 in the ESI†). Thus, an exponential decay appears to be a good approximation for the case of ultrafast ICD of NeKr dimers, and differences between exponential and non-exponential decay behavior are negligible in the present study. This was very different in the ICD of HeHe dimers, which can take more than 10 ps.^28^ In that case a non-exponential character was clearly evident. The exponential decay in the present case can be justified by the fact that the most populated vibrational levels (*i.e.*, those with the highest Franck–Condon factors) have similar lifetimes (between 12 fs and 16 fs), see Fig. 2(b). Since the lifetime broadening of the vibrational states is greater than the energetic difference between them (not shown here), they cannot be considered as independently decaying states. This explains the difference between the individual vibrational lifetimes and the total ICD lifetime, which was obtained from an exponential fit to the squared norm of the decaying state wave packet.

**Fig. 6:**
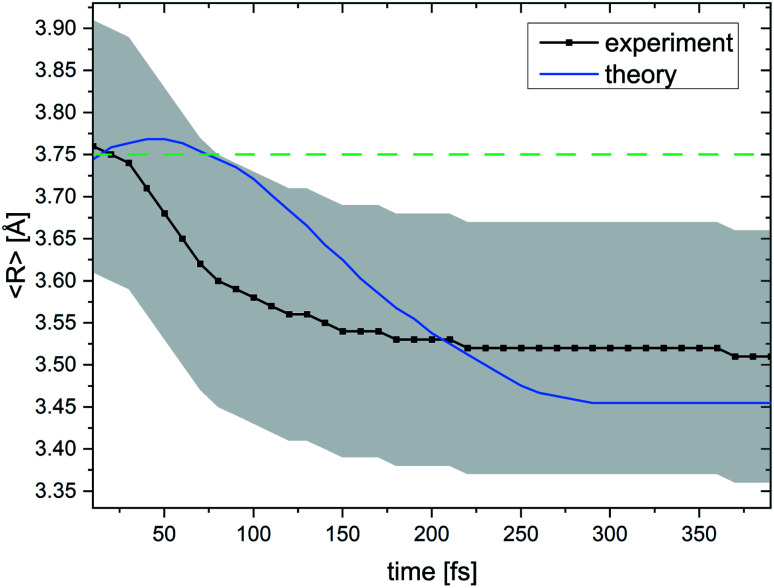
Mean internuclear distance *R* in the Ne^+^ + Kr^+^ breakup of the (NeKr)^++^ dimer dication in the ICD process as a function of the decay time. For every decay time the black curve shows the maximum of a Gaussian function that was used to fit the experimental *R* data (see Fig. 4 for certain decay times). The blue curve shows the theoretical results. A contraction of the NeKr dimer from 3.76 Å to 3.51 Å is clearly visible in the experimental data. Again, in this range only the dissociating Coulombic PECs play a role. The error in the experimental *R* is estimated to be ±0.15 Å, which is represented by the grey shaded area. For the 48.68 eV experiment, we were able to extract decay times down to 10 fs. The green dashed line at 3.75 Å shows the mean internuclear distance of the neutral NeKr dimer, computed from the ground-state wave packet density distribution.

Fig. 6 summarizes our findings on the temporal evolution of the dynamics of interatomic Coulombic decay in NeKr dimers. It depicts the mean internuclear distance as a function of the decay time of the Ne^+^(2s^−1^)Kr dimer cation as retrieved from the two measurements (black line) and from our theoretical modeling (blue line). The theoretical mean internuclear distance was computed similarly to the experimental one, *i.e.*, as the fitted maximum of the internuclear-distance distribution as a function of the decay time, as shown in Fig. 4(b). As can be seen in Fig. 6, the agreement between the theoretical and the experimental mean distance is satisfactory, as the computed mean distance is within the experimental error. It appears that the entire wave packet already decayed before the inner turning point of the attractive potential was reached [see black PEC in Fig. 1(a)]. Otherwise we would expect to observe vibrational revivals in the KER, as seen in the ICD dynamics of the HeHe dimers.^28,68^

## Conclusions

We have extended our experimental PCI-streaking approach to resolve ICD in dimers in order to time ultrafast decay dynamics. In contrast to our previous studies on the evolution of ICD in the time domain in homonuclear HeHe dimers,^28^ which took place on picosecond (ps) time scales, in this work we could successfully track the dynamics in heteronuclear NeKr dimers in the 1000 times faster femtosecond time domain. Our applied electron-streaking scheme is based on the time-dependent post-collision interaction between a very slow photoelectron and a fast ICD electron (which “overtakes” the photoelectron). This measurement scheme could resolve nuclear dynamics on the femtosecond time scale even though single synchrotron light pulses with 80 ps duration were applied to photoionize the dimer. The results provide insight into the ultrafast time evolution of the vibrational wave packet dynamics of an excited NeKr dimer cation undergoing ICD, and we are able to present snapshots of a “molecular movie”. In general, the ultrafast process of ICD in dimers is thought of as a non-exponential decay (see ref. 28 for another ICD example). In the present study of ICD in NeKr dimers, which happens on very short time scales, the differences between exponential and non-exponential behavior are, however, very small. Therefore, an exponential decay can be used as a good approximation to fit the experimental data and to provide insightful information about the electron recapture process, which are both crucial for the comparison of the measured recapture-corrected data and theoretical results.

We have experimentally shown how ICD in NeKr evolves within the first 250 femtoseconds after photoionization. A time resolution of ∼10 fs is achieved, while changes in the internuclear distance as small as 0.3 Å are tracked. The ICD in NeKr dimers is essentially over after 250 fs. During this time the NeKr dimer cation contracts from 3.76 Å (±0.15 Å) towards the classical turning point of the Ne^+^(2s^−1^)Kr cation potential energy curve, reaching a minimum internuclear distance of 3.51 Å (±0.15 Å), before the ICD electron is released and the dimer is Coulomb-exploding, *i.e.*, the ICD process is complete. Following photo-chemical reactions and relaxations on such ultrafast time scales with such exquisite resolution and differential insight was identified as a grand challenge over a decade ago. It is remarkable that this is now realized at a third-generation synchrotron ring rather than at a femtosecond laser system or free-electron laser. Building on this work, more complex molecular targets with internal degrees of freedom such as bending and structural changes (*e.g.*, isomerization, ring breaking, *etc.*) can be targeted in the future.

In principle, the internal clocking procedure presented here, which does not require a two-pulse pump-probe interrogation scheme, can be applied to investigate other dissociation processes and systems in the time domain. For instance, while employing sophisticated multi-coincidence COLTRIMS spectrometers, not only the slow photoelectron in coincidence with the Coulomb-exploding molecular fragments could be detected, but also the fast ICD (or Auger) electron. The photoelectron, which is decelerated *via* PCI, would again set up the clockwork. The fast electron could be investigated in the molecular frame and in a time-resolved fashion with that very same clock. Auger decays in prototypical small molecules like CO, N_2_, O_2_, or CO_2_ are even faster than ICD in dimers. The time scale of the Auger process amounts to only a few femtoseconds. This means, if successful, that the PCI-streaking method would even open the door to the attosecond time domain.^69^ Furthermore, the combination of suited COLTRIMS setups and hard X-ray beamlines would, for example, enable time-resolved studies of Auger decays of vacancies Xe(1s^−1^) in xenon atoms or I(1s^−1^) in iodine-containing molecules *via* PCI-streaking. This would take our established method deep into the attosecond regime (Xe(1s) core-hole lifetime is ∼68 as, see ref. 70). Based on our current results, it is conceivable to trace the evolution of a hole created inside inner shells of atoms or molecules like, *e.g.*, the hopping of a core hole in molecular nitrogen after inner-shell ionization followed by Auger decay.^71^ Moreover, nuclear conformation changes such as the so-called umbrella motion in the dissociation of NH_3_ after N(1s) photoionization and Auger decay^72,73^ would be traceable in real-time using the same approach.

## Data availability

The datasets generated during the current study are available from the corresponding authors upon reasonable request.

## Author contributions

F. T., R. D., and T. J. designed the experiments and analyzed the data. T. M., N. S., V. S., and K. G. performed the theoretical calculations and modeling. M. W., A. H., M. R., J. B. W., A. G., B. G., J. S., A. L. L., B. B., I. B.-I., T. J., and T. W. conducted the beam time and acquired the experimental data. F. T., T. M., T. J., and T. W. wrote the manuscript and the ESI† with significant review and editing by I. B.-I., N. S., K. G., and R. D. F. T. and T. M. created the figures.

## Conflicts of interest

There are no conflicts to declare.

## Supplementary Material

SC-013-D1SC04630F-s001
