# Supplementary material for: Ultrafast temporal evolution of interatomic Coulombic decay in NeKr dimers
Source: Chem Sci. 2021 Dec 29;13(6):1789–800. doi: 10.1039/d1sc04630f (PMC8827086; doi:10.1039/d1sc04630f)
Supplement: SC-013-D1SC04630F-s001 [file SC-013-D1SC04630F-s001.pdf]

Electronic Supplementary Material (ESI) for Chemical Science.
This journal is © The Royal Society of Chemistry 2022

Ultrafast Temporal Evolution of Interatomic Coulombic Decay in NeKr Dimers

F. Trinter[1,2,*], T. Miteva[3], M. Weller[1,4], A. Hartung[1], M. Richter[1], J. B. Williams[5], A. Gatton[4,6], B. Gaire[4], J. Sartor[6], A. L. Landers[6], B. Berry[7], I. Ben-Itzhak[7], N. Sisourat[3], V. Stumpf[8], K. Gokhberg[8], R. Dörner[1], T. Jahnke[9,†] and Th. Weber[4‡]

[1] *Institut für Kernphysik, Goethe-Universität, 60438 Frankfurt am Main, Germany*
[2] *Molecular Physics, Fritz-Haber-Institut der Max-Planck-Gesellschaft, 14195 Berlin, Germany*
[3] *Laboratoire de Chimie Physique Matière et Rayonnement, UMR 7614, Sorbonne Université, CNRS, 75005 Paris, France*
[4] *Lawrence Berkeley National Laboratory, Chemical Sciences Division, Berkeley, California 94720, USA*
[5] *Department of Physics, University of Nevada, Reno, Nevada 89557, USA*
[6] *Department of Physics, Auburn University, Auburn, Alabama 36849, USA*
[7] *J. R. Macdonald Laboratory, Department of Physics, Kansas State University, Manhattan, Kansas 66506, USA*
[8] *Theoretische Chemie, Physikalisch-Chemisches Institut, Universität Heidelberg, 69120 Heidelberg, Germany*
[9] *European XFEL GmbH, 22869 Schenefeld, Germany*

Supporting Information

Here, we elaborate in more detail on how the measured photoelectron energy shift is used to time the nuclear dynamics of the intermediate $Ne^{+}(2s^{-1})Kr$ ion (see also [1]). A projection of Fig. 3, shown in the main text, on the decay-time axis (x-axis), integrated over all photoelectron energies, yields the decay time distribution (not shown here). In the next step, this mapping function of photoelectron energy to decay time is applied to the energy correlation diagram in Fig. SI 1, which depicts the Ne^{+} + Kr^{+} + e^{-} yield as a function of the KER and the photoelectron energy, in order to plot the KER as a function of the decay time. The results of this procedure are presented in Fig. SI 2, showing the Ne^{+} + Kr^{+} + e^{-} yield as a function of the KER and the decay time.

Figures SI 1(a) and SI 1(b) show the yield of the Coulomb-exploding $(NeKr)^{++}$ dimer dication measured in coincidence with the photoelectron as a function of the measured KER and the photoelectron energy for the two photon energies of 48.60 eV and 48.68 eV, respectively. This particle energy correlation map, which relates lower KER values to lower electron energies, already provides a glimpse of the molecular dynamics during the ICD process. Using the mapping shown in Fig. 3 in the main text, the particle energies are converted to decay times, and we obtain the KER as function of decay time [see Figs. SI 2(a) and SI 2(b)]. From the latter two spectra we can clearly see that the KER of the dimer evolves towards higher values with increasing decay time. According to the reflection approximation [2], the internuclear distance R = 1/(KER) (in atomic units and valid only for Coulombic PECs, see main text) decreases as a function of real-time, meaning that the singly charged $Ne^{+}(2s^{-1})Kr$ dimer tends to shrink before ICD occurs.

Figures SI 3(a), SI 3(b), and SI 4 show the photoelectron energy and KER distributions, integrated over all decay times, respectively. The KER distributions for both photon energies (black and red curves) in Fig. SI 4 are very similar. There are two known effects which could in principle make the KER depend on the photon energy. The first is the nuclear motion induced by the photoelectron recoil as observed in, e.g., [3]. However, this effect is negligible at the present low energies. The second effect is the electron-energy dependence of the photoelectron recapture process. At very short decay times and low initial photoelectron energies, the deceleration of the photoelectron can exceed its initial kinetic energy and lead to a recapture of the photoelectron [4]. This sets a lower limit on which dynamics can be followed in time in this scheme. The influence of the recapture effect becomes visible in more differential data, but according to Figs. SI 3(a) and SI 3(b) it does not significantly alter the total KER. Figure SI 4 also shows the calculated KER.

In Fig. SI 3, the measured photoelectron energy spectra of the dimer (blue) are compared with the photoelectron energy distribution, measured for the photoionization of the Ne monomer (red). The chemical shift between the Ne $(2s^{-1})$ electron in the NeKr dimer and the atomic Ne is clearly visible, reflecting the fact that the photoelectrons in the dimer are faster than in the atom, because the presence of the Kr atom in the NeKr dimer changes the

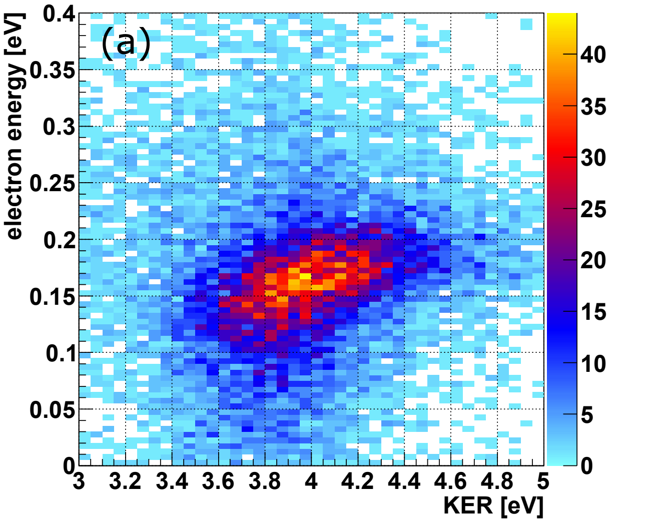

FIG. SI 1: Electron-ion energy correlation map showing the Ne^{+} + Kr^{+} + e^{-} yield as a function of the kinetic energy release and the photoelectron energy for the Coulomb explosion of the $(NeKr)^{++}$ dimer dication, in (a) shown for 48.60 eV photon energy and in (b) for 48.68 eV photon energy.

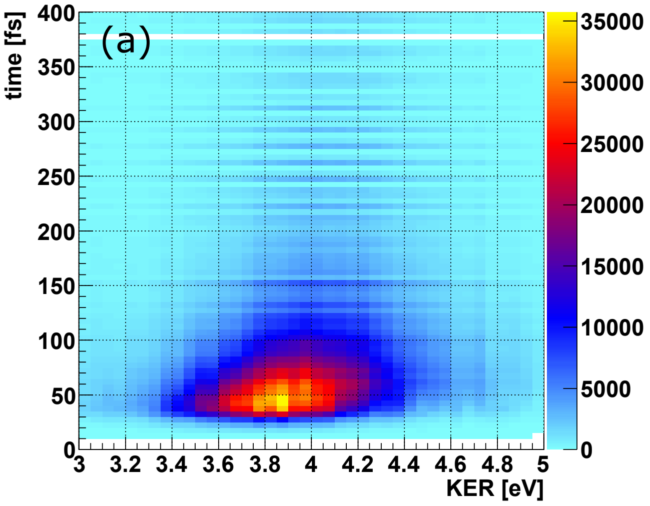

FIG. SI 2: Measured yield of the $(NeKr)^{++}$ dication undergoing Coulomb explosion initiated by the ICD process in NeKr dimers as a function of the kinetic energy release (eV) and the decay time (fs) for (a) 48.60 eV and (b) 48.68 eV photon energy. The yield is derived from the mapping functions based on the spectra presented in Fig. 3 in the main text and Fig. SI 1 (see text for details).

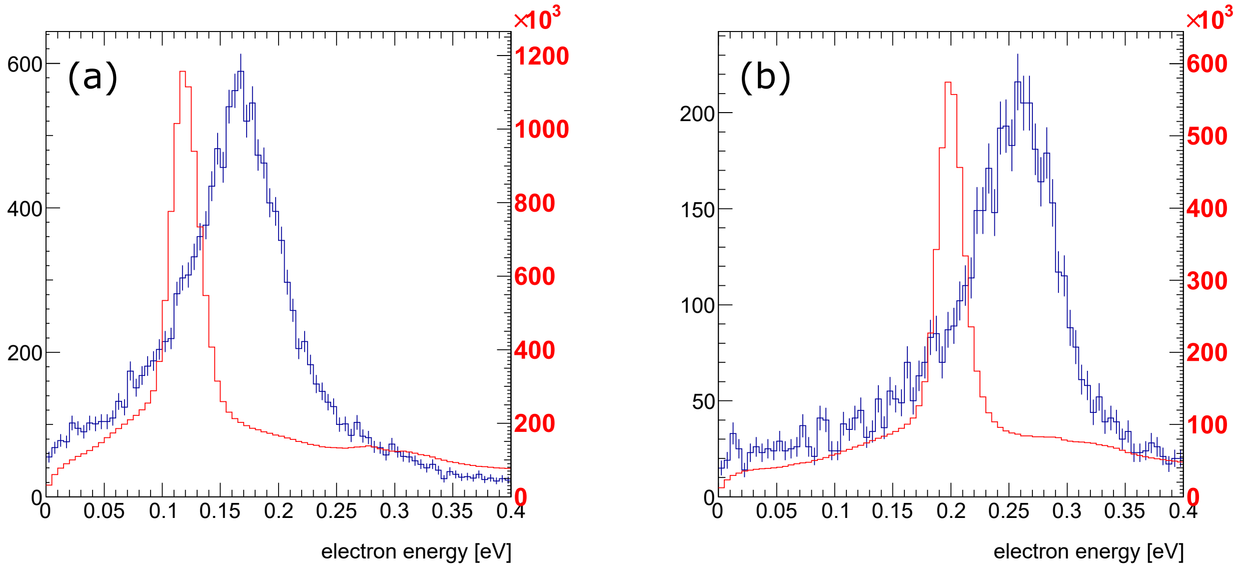

FIG. SI 3: Measured kinetic energy spectra of the photoelectrons released during the ICD process in NeKr dimers. (a) Blue curve: measured photoelectron energy for 48.60 eV photon energy [projection from Fig. SI 1(a)], red curve: 2s line of the Ne monomer. (b) Blue curve: measured photoelectron energy for 48.68 eV photon energy [projection from Fig. SI 1(b)], red curve: 2s line of the Ne monomer.

Ne (2s) binding energy. As the binding energy is lower, the kinetic energy of the electrons in the dimer is higher by approximately 50 meV. The overall shapes of the distributions of the photoelectron energies are very similar for both photon energies. For our method of PCI-streaking to work properly, an ICD electron with significantly higher kinetic energy compared to the photoelectron is needed, because the strength of post-collision interaction depends on the energy difference between the photoelectron and the ICD electron. If ICD electrons energetically overlap with the slow photoelectrons, as it is, e.g., the case in NeNe dimers, where ICD electrons range from 0 eV to roughly 2 eV [5], the deceleration of the photoelectrons is weak or even absent, and, consequently, any ICD time evolution is hard to resolve via PCI-streaking. On the other hand, NeKr dimers are known to produce ICD electrons of around 10 eV [6], and they, hence, represent an ideal species for time-resolved ICD studies employing PCI-streaking.

The mapping procedure described in the main text, which explains how PCI-streaking gives access to tracking the nuclear dynamics of the $Ne^{+}(2s^{-1})Kr$ transient ion in real-time, enables us to finally plot the squared norm of the decaying state wave packet on an ultrafast timescale (see Fig. 5 in the main text). Figure SI 5 represents a zoom of the first 100 fs of the decay time spectrum presented in Fig. 5(b) in the main text, in order to better display the comparison between theory and experiment at ultrashort lifetimes.

* Electronic address: `trinter@atom.uni-frankfurt.de`
† Electronic address: `till.jahnke@xfel.eu`
‡ Electronic address: `tweber@lbl.gov`
[1] F. Trinter, J. B. Williams, M. Weller, M. Waitz, M. Pitzer, J. Voigtsberger, C. Schober, G. Kastirke, C. Müller, C. Goihl, P. Burzynski, F. Wiegandt, T. Bauer, R. Wallauer, H. Sann, A. Kalinin, L. Ph. H. Schmidt, M. Schöffler, N. Sisourat, and T. Jahnke, Evolution of Interatomic Coulombic Decay in the Time Domain, Phys. Rev. Lett. **111**, 093401 (2013).
[2] E. A. Gislason, Series expansions for Franck-Condon factors. I. Linear potential and the reflection approximation, J. Chem. Phys. **58**, 3702 (1973).
[3] K. Kreidi et al., Photo- and Auger-Electron Recoil Induced Dynamics of Interatomic Coulombic Decay, Phys. Rev. Lett. **103**, 033001 (2009).

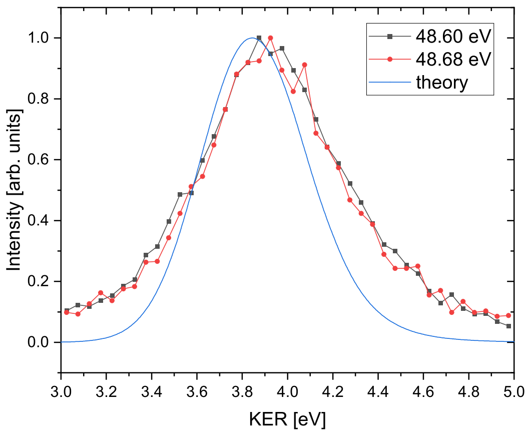

FIG. SI 4: Kinetic energy release (KER) distribution in the Ne^+/Kr^+ breakup of the $(NeKr)^{++}$ dimer dication in the ICD process. Black curve: measured KER for 48.60 eV photon energy, red curve: measured KER for 48.68 eV photon energy, projections from Figs. SI 1(a) and SI 1(b). Blue curve: calculated KER distribution. The differences are within the experimental error bars of ΔKER $\approx \pm 160$ meV.

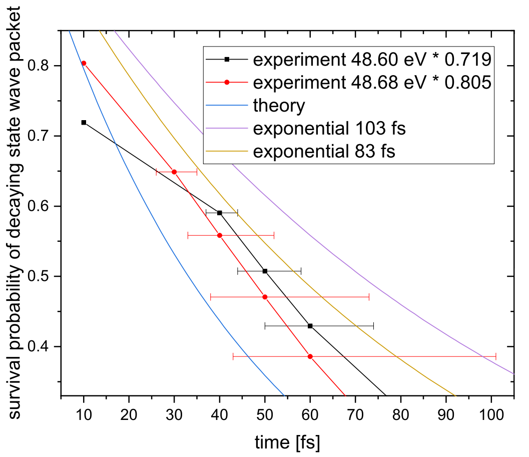

FIG. SI 5: Squared norm of the decaying state wave packet of the intermediate $Ne^+(2s^{-1})$Kr cation state or correspondingly the inverse population of the final state as a function of the decay time. The blue curve represents the theoretical results. The black and red data points depict the experimental results for a photon energy of 48.60 eV (black) and a photon energy of 48.68 eV (red). This spectrum represents a zoom of the first 100 fs decay time. The loss due to the photoelectron recapture is considered. The asymmetric error bars stem from the non-linear conversion of the measured photoelectron energy to the decay time.

[4] P. Burzynski, F. Trinter, J. B. Williams, M. Weller, M. Waitz, M. Pitzer, J. Voigtsberger, C. Schober, G. Kastirke, C. Müller, C. Goihl, F. Wiegandt, R. Wallauer, A. Kalinin, L. Ph. H. Schmidt, M. Schöffler, G. Schiwietz, N. Sisourat, T. Jahnke, and R. Dörner, Interatomic-Coulombic-decay-induced recapture of photoelectrons in helium dimers, Phys. Rev. A **90**, 022515 (2014).
[5] T. Jahnke, A. Czasch, M. S. Schöffler, S. Schössler, A. Knapp, M. Käsz, J. Titze, C. Wimmer, K. Kreidi, R. E. Grisenti, A. Staudte, O. Jagutzki, U. Hergenhahn, H. Schmidt-Böcking, and R. Dörner, Experimental Observation of Interatomic Coulombic Decay in Neon Dimers, Phys. Rev. Lett. **93**, 163401 (2004).
[6] T. Arion, M. Mucke, M. Förstel, A. M. Bradshaw, and U. Hergenhahn, Interatomic Coulombic decay in mixed NeKr clusters, J. Chem. Phys. **134**, 074306 (2011).
